# The dysadherin/FAK axis promotes individual cell migration in colon cancer

**DOI:** 10.7150/ijbs.86699

**Published:** 2024-04-08

**Authors:** Choong-Jae Lee, Tae-Young Jang, Jee-Heun Kim, Songwon Lim, Sunjae Lee, Jeong-Seok Nam

**Affiliations:** School of Life Sciences, Gwangju Institute of Science and Technology, Gwangju 61005, Republic of Korea.

**Keywords:** Dysadherin, Cell motility, Actin dynamics, Focal adhesion, FAK.

## Abstract

Dysregulation of cancer cell motility is a key driver of invasion and metastasis. High dysadherin expression in cancer cells is correlated with invasion and metastasis. Here, we found the molecular mechanism by which dysadherin regulates the migration and invasion of colon cancer (CC). Comprehensive analysis using single-cell RNA sequencing data from CC patients revealed that high dysadherin expression in cells is linked to cell migration-related gene signatures. We confirmed that the deletion of dysadherin in tumor cells hindered local invasion and distant migration using *in vivo* tumor models. In this context, by performing cell morphological analysis, we found that aberrant cell migration resulted from impaired actin dynamics, focal adhesion turnover and protrusive structure formation upon dysadherin expression. Mechanistically, the activation of focal adhesion kinase (FAK) was observed in dysadherin-enriched cells. The dysadherin/FAK axis enhanced cell migration and invasion by activating the FAK downstream cascade, which includes the Rho family of small GTPases. Overall, this study illuminates the role of dysadherin in modulating cancer cell migration by forcing actin dynamics and protrusive structure formation via FAK signaling, indicating that targeting dysadherin may be a potential therapeutic strategy for CC patients.

## Introduction

Cell motility is an essential process for cellular and organismal survival 1-4. This primitive capacity of cells to move has been adapted for homeostasis; however, failure to accomplish this dynamic confers selective growth advantages to cancer cells compared to normal cells in both primary and metastatic colonies. Specifically, metastatic cancer cells hijack normal cellular processes and mechanisms to spread through the body. Colon cancer (CC) ranks third in terms of incidence. CC can be easily managed in the early stage by progressive diagnosis and treatment 5. However, advanced CC remains challenging to treat because it includes cells that have the ability to invade surrounding tissue and migrate to distant sites 6, 7. Therefore, a more comprehensive understanding of the mechanisms of migratory phenotype acquisition is still a requisite for providing therapeutic avenues for advanced CC patients.

Dysadherin is a cell membrane glycoprotein with an FXYD motif that is widely known to be related to metastatic cancer 8. While dysadherin is barely expressed on nonneoplastic cells, abundant expression of dysadherin is frequently observed in various solid cancers, including gastric, colorectal, pancreatic and breast cancers 9-12. Moreover, the degree of clinicopathological features was found to be associated with the range of dysadherin expression in patients 10, 11, 13. In our recent study, genetic depletion of dysadherin attenuated intestinal tumorigenesis in both *Apc*^min/+^mice and AOM/DSS-treated mice 10. However, the fundamental role and mechanism of dysadherin in regulating cell migration, its intermediate molecular mechanism and the consequent pathological results are not yet fully understood.

Here, we characterized the importance of dysadherin for persistent cell migration facilitated by actin dynamics, focal adhesion (FA) turnover and protrusive structure formation. Notably, we observed the involvement of the spontaneous focal adhesion kinase (FAK) signaling cascade in cell motility, thus elucidating the mechanism of dysadherin in motile cells, uncovering a historically dominant view of metastasis, and emphasizing the role of dysadherin as a novel therapeutic strategy for CC.

## Materials and methods

### Cell line and culture

The human CC cell lines HCT116 (KCLB Cat# 10247) and SW480 (KCLB Cat#10228) were purchased from the Korean Cell Line Bank (Seoul, Republic of Korea). All cells were cultured according to the supplier's instructions. Additionally, we used the dysadherin-knockout (KO) SW480 cell line and the overexpression (OE) HCT116 cell line that were established in a previous study 10. The cells were routinely tested for mycoplasma contamination every 6 months using the e-Myco^TM^ Mycoplasma detection kit (iNtron Biotechnology, Seongnam, Republic of Korea), and all experiments were performed within 20 passages from the first thaw.

### Bioinformatic assessment

Gene set enrichment analysis was conducted as described in our previous study 10. Briefly, the differentially expressed gene (DEG) list was obtained from the GSE35896 and GSE21510 datasets by comparing two groups of patients with CC, which were divided according to median dysadherin level. The total DEG list (p < 0.05) was applied as a ranked gene list. Geneset enrichment analysis (GSEA) of the ranked gene list was conducted using the Java implementation of GSEA obtained from http://www.broadinstitute.org/gsea/ (1,000 permutations; minimum term size: 15; maximum term size: 500, C2: all curated genes). The normalized enrichment score accounts for the differences in gene set size. The false discovery rate 1-value was used to set the significance threshold.

For the analysis of single-cell RNA sequencing data, we reanalyzed the datasets GSE132465 and GSE144735 using Seurat for normalization, identification of variable features, scaling, clustering and uniform manifold approximation and projection (UMAP) generation with default parameters. Cell type annotations were imported from the original dataset. Cancer cell samples were classified as dysadherin-high and dysadherin-low based on median dysadherin expression levels. GSEA was performed on pseudo-bulk samples by summarizing all counts from all cells for each gene in the malignant and nonmalignant subsets. Normalization and differential expression analysis were performed using DESeq2, and the R package clusterProfiler was used for pathway enrichment analysis of DEGs.

### *Fxyd5* KO mice

*Fxyd5*^-/-^ mice were established in a previous study 10. Briefly, *Fxyd5*^-/-^ mice were generated using CRISPR/Cas9 technology. Three guide RNAs (gRNA sequences: AGGCTGCTAGGCATCTCGGGGGG, TCTTCCTGGGCTCGGTCACGTGG, and CCCCGATGAGCGATACAGAGACA) were used to cut the genomic DNA at Fxyd5 introns 1 and 7, which resulted in the deletion of exons 2-7, which contain the ATG start codon and most of the coding sequences. To examine the impact of dysadherin deficiency on tumor invasiveness, we developed a murine model by crossing female *Fxyd5*^-/-^ mice with male *Apc*^Min/+^ mice from a C57BL/6J background. *Apc*^Min/+^ mice were purchased from Jackson Laboratory (Bar Harbor, ME, USA). Male mice were sacrificed at 24 weeks of age.

### RNA isolation and real time-qPCR

Total RNA was isolated using RNAiso reagent (Takara, Shiga, Japan). The purity of RNA was verified by measuring the 260/280 and 260/230 absorbance ratios. cDNA templates were synthesized from 0.5 μg of total RNA using the PrimeScript^TM^ 1st strand cDNA Synthesis Kit (Takara Biomedicals, Kusatsu, Japan) with random primers. qPCR was performed using Power SYBR Green PCR Master Mix and Step-One Real-time PCR systems (Applied Biosystems, Foster City, CA, USA). The primers used are listed in Table S1.

### Protein isolation and immunoblotting analysis

Cells were homogenized in radioimmunoprecipitation assay (RIPA) lysis buffer for 20 min on ice. Protein concentrations were determined based on a bicinchoninic acid (BCA) assay using the BCA Protein Assay kit (Thermo Fisher Scientific, Waltham, MA, USA). Proteins were denatured with SDS (Sigma‒Aldrich, St. Louis, MO, USA) by boiling at 95 °C for 5 min. Equal amounts of total protein (4-15 μg) were separated by 8%, 10% or 12% polyacrylamide gel electrophoresis (PAGE), and separated proteins were transferred to a polyvinylidene difluoride membrane (Millipore, Billerica, MA, USA). Membranes were blocked with 5% bovine serum albumin (Sigma‒Aldrich) and incubated overnight at 4 °C with the indicated primary antibodies. Membranes were then incubated with HRP-conjugated secondary antibodies for 1 h. Afterward, membranes were developed using Super Signal West Dura Extended Duration Substrate (Thermo Fisher Scientific) according to the supplier's instructions and analyzed with a FluorChem E System (ProteinSimple, San Jose, CA, USA). Antibodies are listed in Table S2.

### Metastatic mouse model

For the liver metastasis mouse model, empty vector (EV)-transfected control cells or dysadherin KO cells were tagged with luciferase (pCMV-luc) and inoculated into the spleens of NSG mice followed by splenectomy (1 x 10^6^ cells/mouse); surviving cells that grew in distant organs then contributed to the formation of liver metastases. The extent of liver metastasis was routinely monitored weekly by visualizing luciferase activity for 28 days using the IVIS Lumina III *in vivo* Imaging System (PerkinElmer, Waltham, MA, USA). For luciferase detection, 150 mg/mL D-luciferin (PerkinElmer) in PBS was injected intraperitoneally before imaging. After sacrifice, the livers were removed to verify and quantify liver metastasis.

### Histological assessment

For clinical analysis, tissue slides from 20 patients with CC were immunostained by immunofluorescence (IF) to detect dysadherin and p-paxillin using the primary antibody described in Table S2. The slides contained 3 tumor tissue cores and 2 matched normal tissue cores from each patient. The biospecimens and data used for this study were provide by the Biobank fo Chonnam National University Hwasun Hospital, a member of the Korea Biobank Network.

To analyze the liver metastasis mouse model, all liver tissue samples were formalin-fixed and paraffin-embedded within 30 min of removal from mice. Paraffin-embedded tissue blocks were manually sectioned with a microtome to obtain 4-5 μm thick sections. To histologically observe the morphology of tissues and quantify the metastatic burden at the microscopic level, paraffin sections were dewaxed and stained with hematoxylin (Dako, Carpinteria, CA, USA) and eosin (Millipore) according to the supplier's instructions.

In both clinical and mouse tissues, target proteins were visualized by IF. Proteins were visualized using specific antibodies and secondary antibodies conjugated with fluorescent dyes. Nuclei were counterstained with 4′,6-diamidino-2-phenylindole (DAPI, Sigma‒Aldrich). Fluorescence signals were visualized using an Axio Imager 2 (Carl Zeiss, Oberkochen, Germany) at a total magnification of 400x or 1000x. Relative expression levels of the target protein were measured based on fluorescence intensity as described above and normalized to DAPI intensity. Antibodies are listed in Table S2.

### Single-cell tracking

The experimental procedure was performed based on a previously described protocol with modification 14. Briefly, cells were seeded on 35 mm dishes coated with fibronectin. Upon cell adhesion, cells were stained using CellTracker^TM^ Green CMFDA Dye or Red CMTPX Dye (Thermo Fisher Scientific) for 30 min. Then, time-lapse images were captured at a total magnification of 200x in 10 min intervals for 500 min using an FV1000 confocal laser scanning microscope (Olympus, Tokyo, Japan). Time-lapse images were analyzed using IMARIS8 software (BitPlane, South Windsor, CT, USA).

### Knockdown of target genes

Small interfering RNAs (siRNAs) were purchased from Bioneer (Daejeon, Republic of Korea). siRNA transfection was performed using Lipofectamine 2000 (Invitrogen) according to the manufacturer's protocol. Three different siRNA sequences specific for each target were used, and their knockdown efficiencies were measured by RT-qPCR and immunoblotting analysis. The siRNA sequences are listed in Table S3.

### Immunofluorescence staining and quantification

Immunofluorescence assays were conducted as previously described 15. Cells were seeded on poly-L-lysine and collagen I-coated cover glasses and fixed with 4% formalin in phosphate-buffered saline (PBS) at room temperature for 30 min. Cells were permeabilized using 0.3 M glycine and 0.3% Triton X-100, after which they were blocked with 2% normal swine serum (DAKO). Staining was performed using primary antibodies, and secondary antibodies conjugated with fluorescent dyes were used to visualize target proteins (Table S 2). All nuclei were counterstained with DAPI (Sigma‒Aldrich). Fluorescence was visualized using Axio Imager 2 (Carl Zeiss).

For quantitative analysis of protrusion structures, F-actin was stained and visualized with Alexa Fluor^TM^ 555-conjugated phalloidin. Fluorescence intensities of lamellipodia and stress fibers, the density of filopodia and the length of filopodia were measured from images captured at identical settings using an Axio Imager 2 (Carl Zeiss) after background subtraction. To measure the mean fluorescence intensity of the lamellipodia and stress fibers, an ROI of the cell perimeter was made, and the fluorescence intensity was measured from the cell perimeter inward 2.7 µm, which encompassed the lamellipodia and stress fibers 16. The mean intensity along this perimeter was measured and plotted for each condition using ImageJ. To measure the density and length of filopodia, manual tracking and a single image tool were utilized to analyze the filopodia by using the FiloQuant plugin for ImageJ 17. Filopodia density was defined as the ratio of the number of detected filopodia to the cell edge length. Three researchers performed a blind experimental procedure independently in both measurements.

### Transwell assay

The Transwell system (8 μm pore size, Corning, Glendale, AZ, USA) was employed for migration and invasion assays. For the migration assay, 3 x 10^5^ cells were seeded on the upper chambers in serum-free medium. For the invasion assay, 3 x 10^5^ cells were seeded on the upper chamber of a Matrigel-coated Transwell system (8 μm pore size, Corning) in serum-free medium. The bottom chamber was filled with medium supplemented with 5 % fetal bovine serum. After incubation for 24 h at 37 °C, the cells that migrated or invaded through to the bottom of the insert membrane were fixed, stained with crystal violet, and counted under a phase-contrast microscope (Carl Zeiss, biological triplicates).

### RhoA, Rac1 and Cdc42 activation assay

Protein lysates from HCT116 cells were equalized in terms of concentration and assessed for Rho GTPase activation using G-LISA kits (Cytoskeleton, Inc, Denver, CO, USA) specific for the following proteins according to the manufacturer's protocol: RhoA (BK124-S), Rac1 (BK128-S), and Cdc42 (BK127-S). Quantitative analysis of Rho GTPase activation was carried out by measuring the absorbance at a wavelength of 490 nm using Epoch microplate reader (BioTek, Winooski, VT, USA).

### Ethical approval

Prior approval for animal studies was obtained from the Institutional Animal Care and Use Committee (IACUC) of the Gwangju Institute of Science and Technology (GIST, No. GIST2021-065). Analysis of dysadherin and p-paxillin expression in patients with CRC was preapproved by the Institutional Review Board (IRB) at GIST (No. 20200108-BR-50-07-02). All work related to human tissues was conducted in accordance with the Helsinki Declaration. Written informed consent was obtained from all participants prior to the study. *In vitro* experiments were all performed on at least three separate occasions. Exclusion criteria were not applied in this study; thus, outliers were included in all experimental results.

## Results

### Expression of dysadherin can be used to predict CC metastasis

**Figure 1:**
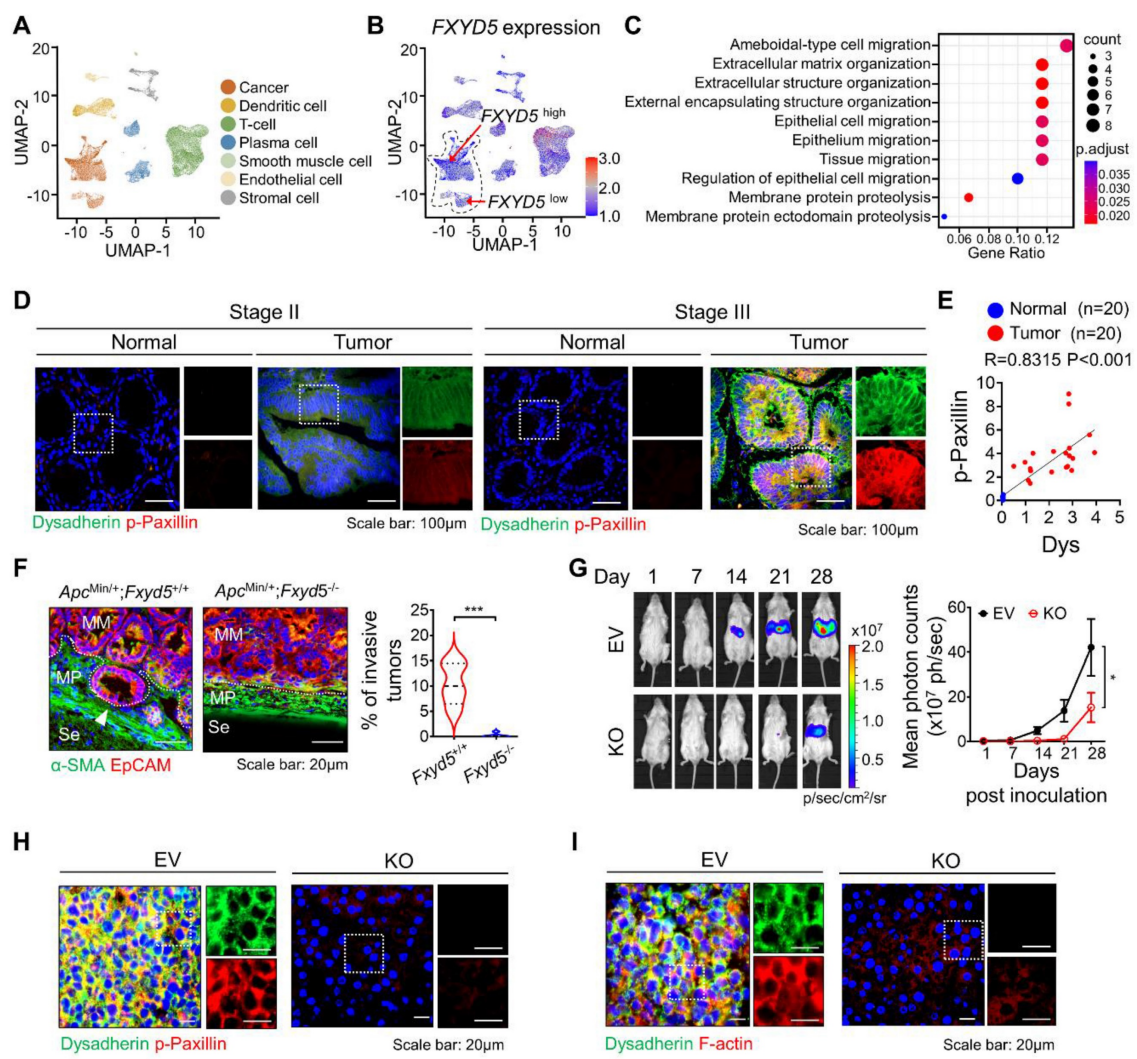
** Dysadherin facilitates the cell migration and invasion, leading to metastasis. (A)** UMAP plot from the tumor tissue of CC patients, showing 7 clusters. Each cluster is shown in a different color. **(B)** Data visualization using a UMAP plot on the basis of dysadherin expression. **(C)** Pathway enrichment analysis using differentially expressed genes in groups of cells with high and low dysadherin expression. **(D)** Immunofluorescence analysis of dysadherin and p-paxillin expression using CRC patient tissue. The patient number used in representative images is 71038481 for stage II and 11841258 for stage III. Scale bars, 100 µm. **(E)** Correlation between dysadherin and p-paxillin in patient samples. **(F)** IF analysis of the intestines of 24-week-old *Apc*^Min/+^ mice labeled for the epithelial marker EpCAM and α-smooth muscle actin (SMA) (n = 5 mice per group). MM: muscularis mucosae; MP: muscularis propria; Se: serosa. The violin plot shows the percentage of invasive tumors among tumors with a diameter over 3 mm. **(G)** Luciferase-labeled dysadherin EV and KO SW480 cells were inoculated into the spleens of NSG mice. Mice were tracked for 28 days after splenic injection (n = 6 mice per group). Representative *in vivo* bioluminescence images (left) of mice injected with luciferase-labeled dysadherin EV and dysadherin KO SW480 cells, accompanied by a corresponding graph showing the quantitative analysis of the region of interest (right). **(H, I)** Immunofluorescence analysis of dysadherin, F-actin and p-paxillin expression in metastatic nodules generated from invaded dysadherin EV or KO SW480 cells. The data are presented as the means ± SEMs. *, **, and *** indicate p < 0.05, p < 0.01 and p < 0.001, respectively. The statistical significance was determined by unpaired two-tailed Student's t test for comparisons between two groups, and differences in tumor growth of mice with liver metastasis were determined by two-way repeated-measures ANOVA followed by the Bonferroni post hoc test.

To verify the role of dysadherin in CC, we analyzed the CC single-cell RNA sequencing (scRNA-seq) dataset (GSE132465 and GSE144735) 18. We clustered cells and assigned the cell type of each cell (Figure 1A). Next, to investigate the effect of dysadherin expression on cancer cells, we characterized tumor cells on the basis of high or low expression of dysadherin (FXYD5) based on the medial gene expression (Figure 1B). After grouping the cells by dysadherin expression, we performed pathway enrichment analysis based on DEGs (adjusted p-values < 1×10^-3^) between the high and low dysadherin groups, which showed that tumor cells with high dysadherin expression exhibited upregulated migration-related gene signatures, including genes involved in ameboidal-type cell migration and epithelial cell migration (hypergeometric tests adjusted p-values < 0.05, Figure 1C and S1A). To gain further insight into the role of dysadherin in colorectal cancer (CRC) patients, we compared the gene expression profiles of tumors by dividing 62 CRC patient samples (GSE35896) and 104 CRC patient samples (GSE21510) into high dysadherin expression (Dys^high^) groups and low dysadherin expression (Dys^low^) groups based on median values. The list of differentially expressed genes in Dys^high^ tumors was subjected to GSEA. As a result, the metastasis gene signature along with the invasion, cell migration, metastasis and epithelial-mesenchymal transition (EMT) gene signatures were significantly enriched in Dys^high^ tumors (Figure S1B and S1C). Consistent with this finding, we assessed the expression of dysadherin in CRC by comparing stage II CRC, which had not spread, and stage III CRC, which had spread to lymph nodes (Table S4 and S5), and verified the correlation between dysadherin and p-paxillin in cell migratory and invasive phenotypes 19. As a result, dysadherin expression was higher in stage III CRC tissue than in stage II CRC tissue, and p-paxillin expression was high in tumor tissue with high dysadherin expression, while p-paxillin expression was low in tumor tissue with low dysadherin expression (Figure 1D and S1D). A positive correlation was observed between dysadherin and p-paxillin expression in patient tissues (Figure 1E). Furthermore, we checked the expression of dysadherin and p-paxillin in carcinoma *in situ* and metastatic CRC including primary tumor and liver metastasis (Table S6 and S7). Expression of dysadherin and p-paxillin was higher in metastatic CRC than in carcinoma *in situ* (Figure S1E). Therefore, we confirmed that high dysadherin expression was positively associated with p-paxillin and metastasis of CRC.

Next, dysadherin-knockout (*Fxyd*5^-/-^) mice were established in a previous study, and we confirmed the role of dysadherin in invasive potential by using a 24-week-old *Apc*^Min/+^ mouse model in which tumors ranged from well-differentiated adenomas to invasive adenocarcinomas 10. In *Apc*^Min/+^ mice, deletion of dysadherin significantly decreased not only the total tumor load but also the number of polyps by size distribution (< 1 mm diameter polyps, 1-3 mm, 3-5 mm and > 5 mm) (Figure S1F). When we analyzed tumors with a diameter of more than 3 mm, approximately 10.4% of tumors invaded through a fissure of the mucosal muscle layer. However, all tumors in *Apc*^Min/+^;*Fxyd5*^-/-^ mice remained above the mucosal muscle layer, showing a noninvasive phenotype (Figures 1F and S1G). Furthermore, we validated the relevance of dysadherin and metastatic potential by performing a splenic injection model, which represented the ability of cells to migrate to distant organs and colonize. Dysadherin KO dramatically decreased the metastatic incidence of CC cells in the liver, indicating that dysadherin plays a critical role in the metastasis of CC cells (Figures 1G, S1H and S1I). In addition, our histological analysis of metastasized livers collected from the splenic injection mouse model revealed that decreased p-paxillin expression in the metastasized liver was significantly correlated with the expression of dysadherin (Figures 1H and S1J). Interestingly, we found a significant reduction in F-actin in dysadherin KO CC-bearing mice, as observed in a previous report (Figures 1I and S1K). Therefore, these data suggest that dysadherin modulates the motility dynamics of individual cells, such as the FA and actin cytoskeleton.

### Dysadherin modulates the motility of individual cells

**Figure 2:**
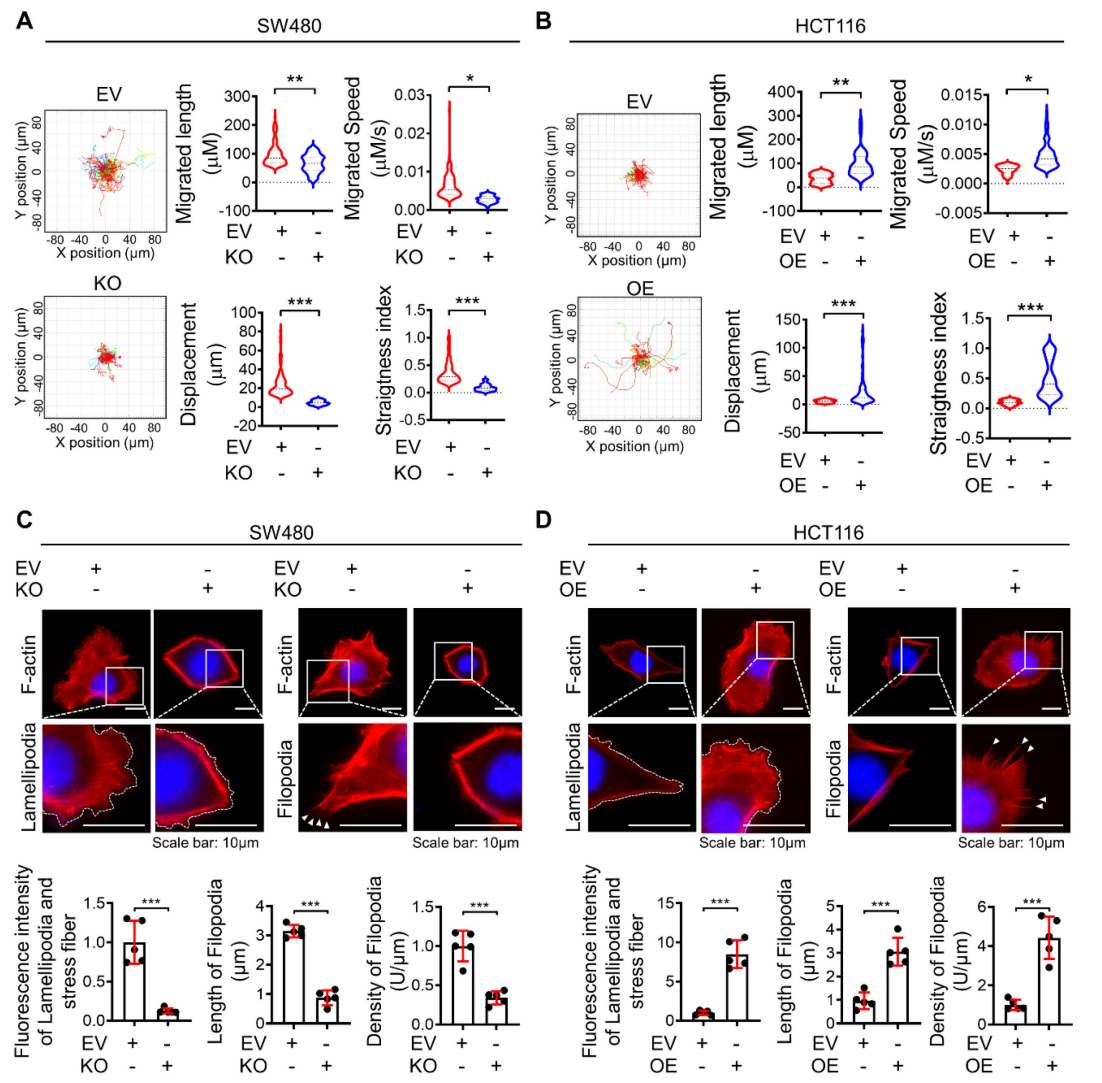
** Differential expression of dysadherin alters single-cell motility and formation of lamellipodia and filopodia. (A)** Wind-rose plot representing trajectories of individual cells (n=100) in a single field of view over an 8 h period for both dysadherin EV and KO SW480 cells. Scatter plots showing the migrated length (µM), migrated speed (µM/s), straightness index and directionality. **(B)** Wind-rose plot representing trajectories of individual cells (n=100) in a single field of view over an 8 h period for both dysadherin EV and OE HCT116 cells. Scatter plots show migrated length (µM), migrated speed (µM/s), straightness index and directionality. **(C)** Immunofluorescence analysis of protrusive structure formation in dysadherin EV and KO SW480 cells. Immunofluorescence staining is shown for F-actin (red) and DAPI staining (blue) with the corresponding magnified images. Scale bars, 10 µm. Graphs (bottom) show the fluorescence intensity of lamellipodia and stress fibers, density of filopodia and length of filopodia. **(D)** Immunofluorescence analysis of protrusive structure formation in dysadherin EV and OE HCT116 cells. Immunofluorescence staining is shown for F-actin (red) and DAPI staining (blue) with the corresponding magnified images. Scale bars, 10 µm. Graphs (bottom) show the fluorescence intensity of lamellipodia and stress fibers, density of filopodia and length of filopodia. The data are presented as the means ± SEMs. *, **, and *** indicate p < 0.05, p < 0.01 and p < 0.001, respectively. The statistical significance was determined by unpaired two-tailed Student's t test for comparisons between two groups.

It is well established that hyperactivated motile behaviors of individual cells are fundamental for metastatic potential. Therefore, to obtain deeper insight into the biological role of dysadherin in single-cell motility, we performed live-cell imaging and analyzed the trajectories of individual cells by monitoring cells for over 8 h. Trajectories and motile behaviors were evaluated with various parameters, e.g., migrated length, migrated speed, straightness index (refers directionality) and displacement. First, dysadherin-depleted cells remained rather tethered in place without persistent motion beyond the original cell borders, as well as definite lower average directionality compared to control cells (Figure 2A, Videos S1, 2). Conversely, the OE of dysadherin (Figure S1L) significantly enhanced migration length, speed, displacement and directionality (Figure 2B, Videos S3, 4). Collectively, these results clearly indicate that dysadherin is a key factor that modulates the motile behaviors of individual cells.

### Dysadherin formulates actin-based protrusion structures of individual cells

For the first event of cell migration, actin-based protrusions are generated for each cell to be ready to crawl. These protrusions are driven by the polymerization of actin filament arrays and often take on a flatter appearance and fan-like architecture (referred to as 'lamellipodia) as well as a linear microspike-like architecture (referred to as 'filopodia'). Of note, these different protrusions are not finite entities; they are highly plastic and can dynamically interconvert over time, such as when filopodia direct where lamellipodia can form or when lamellipodia yield clusters of filopodia. Therefore, the proportion of the actin network within protrusions is probably more important than the naming conventions of the protrusion type. In this context, to confirm the effect of dysadherin on single-cell motility at the initiating stage of cell movement, we evaluated the intensity of lamellipodia and stress fibers, the density of filopodia and the length of filopodia based on F-actin staining upon genetic alteration of dysadherin. KO of dysadherin led to significantly fewer protrusion structures, which was also quantified and represented by a lower intensity of lamellipodia and stress fibers along with shorter but sparse filopodia formation (Figure 2C). In contrast, OE of dysadherin led to more protrusion structures, which was supported by the higher intensity of lamellipodia and stress fibers with longer but condensed filopodia generation (Figure 2D).

### Dysadherin is responsible for regulating the dynamic molecular signaling continuum of cell motility

**Figure 3:**
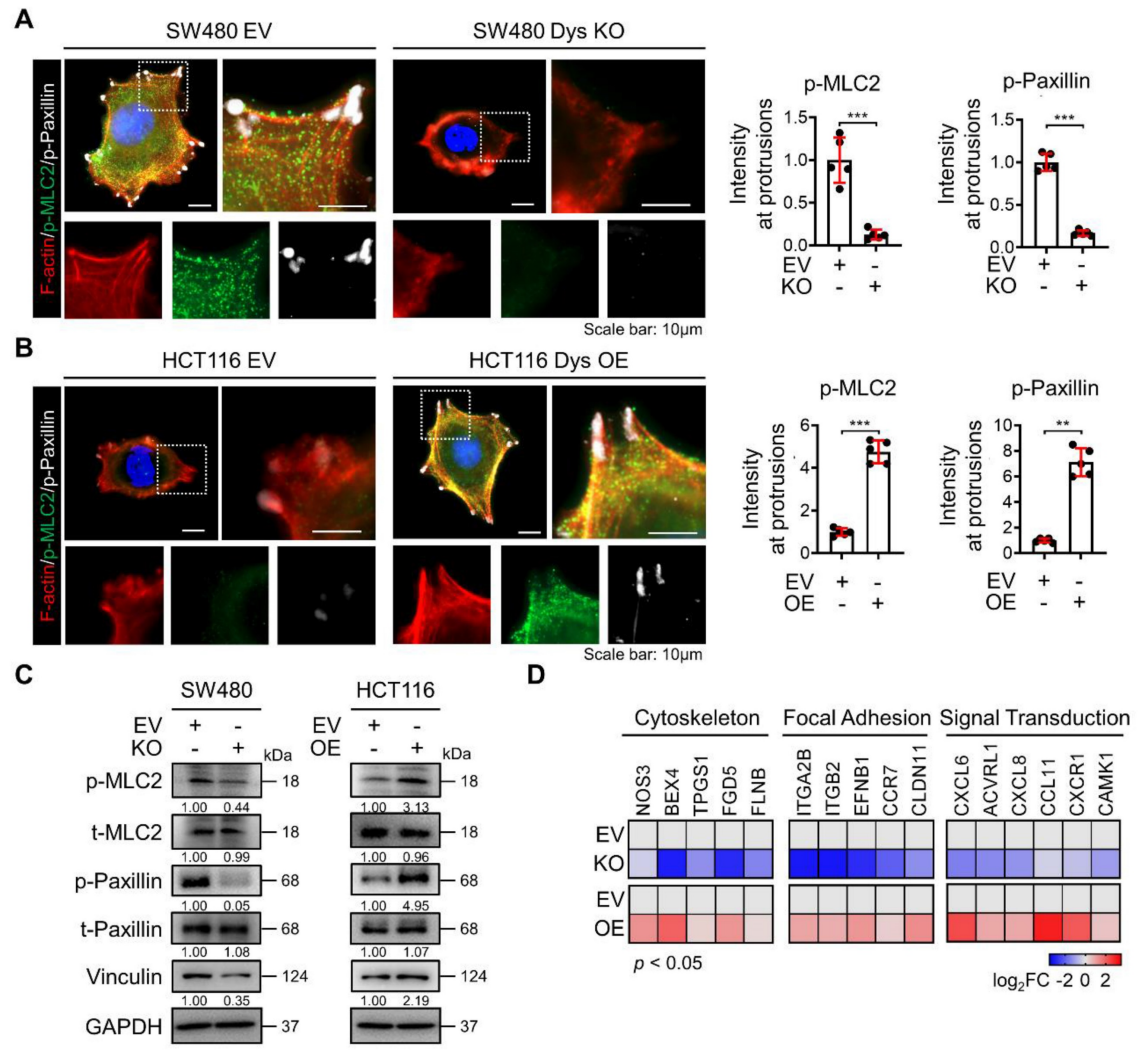
** Dysadherin is responsible for the regulation of the dynamic molecular signaling continuum of cell motility. (A, B)** Immunofluorescence analysis of F-actin, p-MLC2 and p-paxillin in dysadherin EV and KO SW480 cells **(A)** and in in dysadherin EV and OE HCT116 cells **(B)**. Immunofluorescence staining is shown for F-actin (Red) p-MLC2 (green), p-paxillin (white) and DAPI staining (blue). Scale bars, 10 µm. Graphs show the mean fluorescence intensity (MFI) of p-MLC2 and p-paxillin at protrusions. **(C)** Immunoblot of p-MLC2, t-MLC2, p-paxillin, t-paxillin, and vinculin upon the alteration of dysadherin in SW480 and HCT116 cells. **(D)** Heatmap comparing the relative expression of cytoskeleton, FA and signal transduction-related genes in dysadherin EV and KO SW480 and EV and OE HCT116 cells, as determined by RT‒qPCR (n = 3 biological replicates). The data are presented as the means ± SEMs. *, **, and *** indicate p < 0.05, p < 0.01 and p < 0.001, respectively. The statistical significance was determined by unpaired two-tailed Student's t test for comparisons between two groups.

After cells successfully generate protrusion structures, these structures are then stabilized by integrin-based protein complexes known as FAs that connect the actin cytoskeleton to the extracellular matrix (ECM). Then, cells generate traction forces at the adhesion sites and release adhesion at the rear, which allows the cell to move forward 20, 21. Therefore, this sequence of events involves a dynamic organization of the actin cytoskeleton and a controlled assembly and disassembly of FA that may be coordinated spatiotemporally. To investigate the role of dysadherin in the whole process of regulating the dynamic continuum of cell motility, we first investigated the effect of genetic alteration of dysadherin on actin polymerization and FA formation by observing the phosphorylation of myosin light chain 2 (MLC2) and paxillin co-stained with F-actin. As a previous report revealed that the ECM-integrin pathway is a key dysadherin signaling pathway 10, we observed that dysadherin KO significantly reduced levels of p-paxillin and p-MCL2 in protrusion region as indicated by F-actin, representing a reduction in FA formation and actin polymerization, respectively (Figure 3A). In this context, OE of dysadherin increased FA formation and actin polymerization by promoting paxillin and MLC2 phosphorylation at protrusion regions (Figure 3B). Moreover, vinculin, an actin-binding FA protein that is also known to contribute to FA-mediated mechanotransduction, was also altered by dysadherin (Figure 3C). Furthermore, transcriptional analysis showed that dysadherin KO led to a global decrease in cytoskeleton-, FA-, and signal transduction-related gene expression, while dysadherin OE led to an increase in their expression (Figure 3D). Collectively, these results support the idea that dysadherin is responsible for regulating a dynamic molecular signaling continuum related to cell motility.

### Inhibition of FAK signaling attenuates dysadherin-induced persistent cell motility

**Figure 4:**
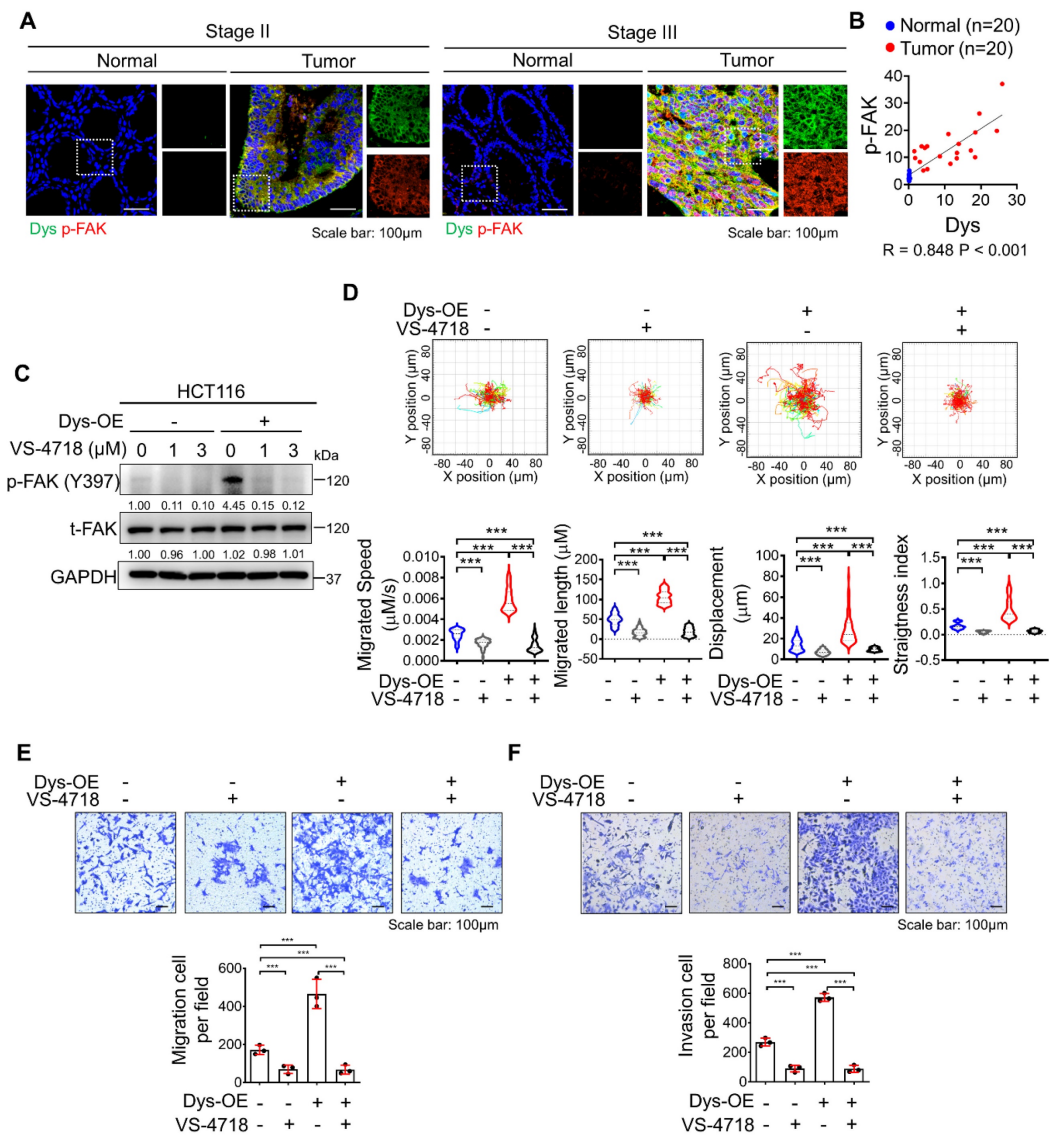
** Inhibition of FAK signaling halts dysadherin-induced cell motility. (A)** Immunofluorescence analysis of dysadherin and p-FAK expression in CRC patient tissue. The patient number used in representative images is 71023619 for stage II and 71047123 for stage III. Scale bars, 100 µm. **(B)** Correlation between dysadherin and p-FAK in patient tissue. **(C)** Immunoblot of p-FAK and t-FAK in dysadherin WT HCT116 and dysadherin OE HCT116 cells with or without VS-4718 treatment. **(D)** Trajectories of individual cells (n=100) in a single field of view over an 8 h period upon 1 µM VS-4718 treatment. Scatter plots showing the migrated length (µM), migrated speed (µM/s), straightness index and directionality. **(E)** Boyden chamber assays without Matrigel matrix-coated membranes were performed to compare the chemotactic migration potential of dysadherin WT HCT116 and dysadherin OE HCT116 cells with or without 1 µM VS-4718 (n = 3/group). **(F)** Boyden chamber assays with Matrigel matrix-coated membranes were performed to compare the invasion potential of dysadherin WT HCT116 and dysadherin OE HCT116 cells with or without 1 µM VS-4718 (n = 3/group). The data are presented as the means ± SEMs. *, **, and *** indicate p < 0.05, p < 0.01 and p < 0.001, respectively. The statistical significance was determined by unpaired two-tailed Student's t test for comparisons between two groups.

In a previous report, we demonstrated that the dysadherin-fibronectin-integrin-FAK axis is responsible for generating mechanical force, which provides a beneficial environment for activating downstream biochemical signals such as FA assembly and yes-associated protein 1, thus facilitating intestinal tumorigenesis and the progression of malignancy 10, 22, 23. In this study, we investigated the role of dysadherin-mediated FAK activation in cell motility. We verified the expression of dysadherin and p-FAK in stage II CRC patients and stage III CRC patients. As a result, p-FAK expression was high in tumor tissue with high dysadherin expression, while p-FAK expression was low in tumor tissue with low dysadherin expression (Figures 4A and S2A); furthermore, a positive correlation was observed between dysadherin and p-FAK expression in patient tissues (Figure 4B). We also checked the expression of dysadherin and p-FAK in carcinoma *in situ* and metastatic CRC (Table S6 and S7). Expression of dysadherin and p-FAK was higher in metastatic CRC than in carcinoma *in situ* (Figure S2B). Therefore, we confirmed that high dysadherin expression was positively associated with expression of p-FAK as well as metastasis of CRC. Next, we assessed whether FAK inhibition affects dysadherin-mediated cell motility using VS-4718, a FAK inhibitor, and estimated the half-maximal inhibitory concentration (IC_50_) value of VS-4718 to evaluate its functional effect on cell motility at levels that had only minimal cytotoxic effects. The IC_50_ value was approximately 7.194 μM in HCT116 cells (Figure S2C). VS-4718 treatment led to a reduction in the phosphorylation of FAK at 1 μM and 3 μM, however the difference was not significant. In addition, the phosphorylation of FAK was increased in dysadherin OE cells, and VS-4718 diminished dysadherin-induced FAK activation (Figure 4C). Therefore, we used 1 μM in further experiments and observed that the VS-4718-mediated inhibition of FAK significantly reduced dysadherin-induced cell motility. To confirm the dependence of FAK signaling on dysadherin-induced individual cell motility, we monitored the individual cell motile behavior of dysadherin OE cells under VS-4718 treatment; reduced single-cell motility was observed upon VS-4718 treatment (Figure 4D, Video S5-8). Next, we further verified the dependence of FAK signaling on dysadherin-mediated cell migration by migration and invasion assays. Cell migration and invasion were significantly increased upon OE of dysadherin, and they were reduced upon VS-4718 treatment (Figure 4E and 4F).

### Dysadherin modulates the dynamics of FA through the FAK/Rho GTPase axis

**Figure 5:**
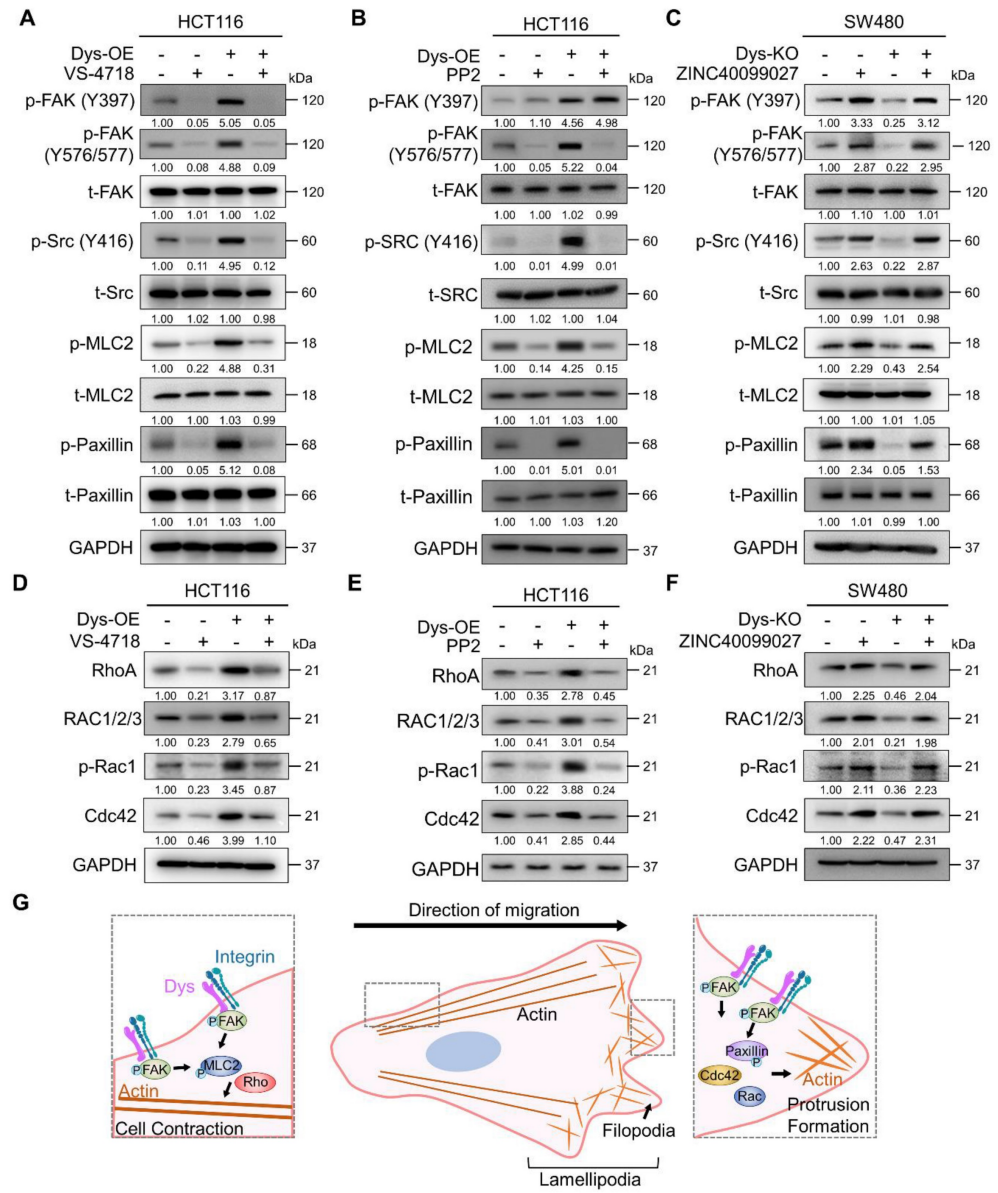
** Dysadherin reorganizes the actin cytoskeleton via FAK and its downstream signaling. (A-C)** Immunoblot of p-FAK, t-FAK, p-Src, t-Src, p-MLC2, t-MLC2, p-paxillin, and t-paxillin in dysadherin EV and OE HCT116 cells with or without 1 µM VS-4718 or 2.5 µM PP2 treatment and in EV and dysadherin KO SW480 cells with or without 10 nM ZINC40099027 treatment. **(D-F)** Alteration of Rho-GTPase proteins upon expression regulation of dysadherin and FAK inhibition using dysadherin EV and OE HCT116 cell line with or without 1 µM VS-4718 or 2.5 µM PP2 treatment and in EV and dysadherin KO SW480 cell lines treated with the FAK activator ZINC40099027 at 10 nM. **(G)** Schematic summary of the study findings, indicating the role of dysadherin in cancer cell migration. The data are presented as the means ± SEMs. *, **, and *** indicate p < 0.05, p < 0.01 and p < 0.001, respectively. The statistical significance was determined by unpaired two-tailed Student's t test for comparisons between two groups.

FAK is autophosphorylated at residue Y397 by the dysadherin-fibronectin-integrin axis 10. When FAK undergoes autophosphorylation, it interacts with Src, which leads to a phosphorylation cascade, resulting in the phosphorylation of FAK at residue Y576/577 in the kinase domain 24-28. So, to verify dysadherin-mediated FAK-Src phosphorylation and its downstream signaling, we performed an immunoblot assay investigating both the levels of total and phosphorylated FAK, Src, MLC2 and paxillin. First, we tested the effectiveness of siRNA targeting FAK (siFAK) and chose the most effective sequence #3 (Figure S3A). Dysadherin OE cells showed elevated levels of phosphorylation of each FAK signaling component but no alteration in the amount of the total form; we further confirmed that dysadherin-induced signaling activation is dependent on FAK activation by treating cells with VS-4718 or siFAK (Figures 5A and S3B). Additionally, dysadherin OE upregulated the expression of FAK downstream genes, but it was reversed by FAK knockdown (Figure S3C). Next, we checked the phosphorylation status of FAK, Src and downstream signaling proteins using the Src inhibitor PP2. We assessed the IC_50_ value of PP2 to evaluate its cytotoxic effects, and the IC_50_ value was approximately 4.86 μM in HCT116 cells (Figure S3D). Therefore, we treated cells with 2.5 μM PP2, a noncytotoxic concentration. The phosphorylation of other FAK signaling components was increased upon dysadherin OE, while treatment with PP2 reduced the levels of p-FAK (Y576/577) and downstream proteins except for p-FAK (Y397) (Figure 5B). We further verified the dysadherin/FAK axis and its downstream signaling using the FAK activator ZINC40099027 29, 30. We assessed the IC_50_ value of ZINC40099027 to evaluate cytotoxic effects, and there was no cytotoxic effect (Figure S3E). Therefore, we used 10 nM in further experiments, which was the concentration used in a previous study 29, 30, and observed the ZINC40099027-mediated activation of FAK and its downstream molecules. The KO cells showed a reduced level of phosphorylation of FAK, Src, MLC2 and paxillin, which was elevated upon FAK activation by ZINC40099027 (Figure 5C). Next, we further verified the dependence of FAK signaling on dysadherin-mediated cell migration by single cell tracking assay and invasion assays. Cell migration and invasion were significantly reduced upon KO of dysadherin, and they were increased upon ZINC40099027 treatment (Figure S3F and S3G).

Direct physical crosslinks or dynamic behavior of cytoskeletal filaments are known to be regulated by Rho GTPases, which are downstream proteins of FAK 31-33. We conducted an immunoblot assay to verify whether the protein levels of Rho-GTPases are reliant on dysadherin expression and FAK signaling status. As a result, OE of dysadherin increased the protein levels of Rho-GTPase proteins, e.g., RhoA, Rac1/2/3, p-Rac1 and Cdc42, whereas VS-4718, siFAK or PP2 treatment diminished the dysadherin-induced expression of Rho-GTPase proteins (Figures 5D, 5E and S3H). Dysadherin KO caused a decrease in the protein levels of Rho-GTPase proteins, but this decrease was reversed by treatment with ZINC40099027 (Figure 5F).

To comprehensively investigate the mechanisms by which FAK phosphorylation regulates the protein levels of Rho-GTPases, we performed a pathway analysis using ingenuity pathway analysis. Phosphorylated FAK causes the activation of various signaling pathways, such as the Akt, ERK1/2, and Src pathways, and increases the expression of Rho-GTPase proteins (Figure S3I). Furthermore, FAK phosphorylates paxillin through direct binding, which activates several GTPase-activating proteins and guanine nucleotide exchange factors, thereby controlling the activity of the Rho-GTPase proteins (Figure S3J). To verify the activation status of Rho-GTPase proteins by dysadherin-FAK-paxillin, we performed RhoA, Rac1 and Cdc42 activation assays to verify the phosphorylation status of Rho-GTPase proteins. We tested the effectiveness of siRNA targeting paxillin and chose the most effective sequence, #2 (Figure S3K). The phosphorylated forms of Rho-GTPase proteins were increased in dysadherin OE cells, while paxillin knockdown reversed the dysadherin OE-induced phosphorylation of Rho-GTPase proteins (Figure S3L). In addition, treatment with VS-4718 also reduced the dysadherin OE-induced phosphorylation of Rho-GTPase proteins (Figure S3M). Taken together, these results indicate the dependence of dysadherin on the FAK signaling axis, particularly in regulating cell motility (Figure 5G).

## Discussion

This study revealed the role of dysadherin in regulating the migration and invasion of CC cells by performing comprehensive analysis. We identified for the first time that high dysadherin expression in cells is associated with cell migration-related gene signatures by analyzing single-cell genomics studies. Dysadherin expression is associated with migratory and invasive phenotypes in mouse models and CRC patient samples. Of note, genetic depletion of dysadherin in tumor cells impeded invasion into surrounding tissues and migration to distant organs using *in vivo* tumor models. Consistently, dysadherin was found to play a critical role in single-cell migration of CC cells through single-cell tracking and morphological analysis. Mechanistically, we identified dysadherin as a regulator of actin dynamics and protrusive structure formation via FAK and its downstream signaling. Therefore, our findings are an important step toward unraveling the functional importance of dysadherin in regulating cancer cell migration.

Seeking to understand how dysadherin modulates these cytoskeleton dynamics, we further investigated the underlying molecular mechanisms. Numerous studies have established FAK as an important component of signaling mediated by other cell surface receptors in many cell types that contribute to the pathogenesis of cancer and other diseases 34, 35. As an intracellular protein-tyrosine kinase (PTK) recruited to and activated at FA sites, FAK is known to be a key signaling PTK that acts downstream of various growth factors and ECM components 36. In parallel to these known activities, our recent study also showed the relevance of the dysadherin-fibronectin interaction in sustaining fibronectin-integrin-FAK axis activation 10. Moreover, activated FAK is known to recruit c-Src at FA sites to form a FAK-Src signaling complex; therefore, this complex phosphorylates other FA signaling and adapter proteins, such as paxillin, another important cytoskeletal and scaffolding protein that is recruited early to nascent FAs at the cell front and is necessary for FA turnover, thereby activating diverse signaling pathways in the regulation of cell motility 37. Herein, our study suggests the role of paxillin in regulating myosin-light-chain-kinase-dependent contractility in the FAK-Src complex, which clearly shows the role of paxillin in FA assembly but may also provide a hint about the precise mechanism controlling adhesion disassembly.

In this study, genetic alteration of dysadherin regulated the expression of the Rho family downstream of FAK signaling. This is the first study to show that dysadherin regulates actin dynamics and protrusive structure formation via the FAK/Rho family axis. The Rho family of small GTPases is well known to facilitate the dynamic behavior of the actin cytoskeleton and stress fibers by stimulating the assembly of contractile actin arrays or by promoting the assembly of protrusive arrays 38-40. Generally, Cdc42 functions to regulate cell polarity and the formation of filopodia, while Rac1 mediates the formation of lamellipodia at the leading edge of cells. RhoA is involved in the actomycin contraction and retraction of cells. Our findings suggest that dysadherin controls overall individual cell motility, such as front-rear cell polarization and cell contraction through regulation of Rho family activity 39-41. In this context, further investigation of the relevance of dysadherin and FAK-mediated Rho GTPases is indispensable to be able to determine the physical principles and their relevance to subsequent biochemical signaling of actin dynamics in terms of inducing cell motility in more detail.

Moreover, dysadherin is a glycoprotein, as it possesses an extended extracellular O-glycosylated domain. Previous study has shown that inhibiting O-glycosylation by incubating cells with benzyl 2-acetamido-2-deoxy-α-d-galactopyranoside (benzyl-α-GalNAc) decreases the stable expression of dysadherin and weakens the effects of dysadherin on cell adhesion and morphology 8, 42. Based on previous study, we verified the effect of inhibiting glycosylation on cell migration and invasion regulated by dysadherin. First, benzyl-α-GalNAc had no cytotoxic effect (Figure S4A). Next, the expression of dysadherin was increased in dysadherin OE cells and significantly reduced after benzyl-α-GalNAc treatment (Figure S4B). Downregulation of dysadherin was dependent on the concentration of benzyl-α-GalNAc (Figure S4C). In addition, we confirmed motility and invasion ability by single-cell tracking and Transwell invasion assay. Single-cell motility and cell invasion were significantly increased upon dysadherin OE, and these changes were reversed upon benzyl-α-GalNAc treatment (Figure S4D and S4E, Videos S9-12). Therefore, glycosylation plays an important role in the stable expression of dysadherin as well as cell migration and invasion. Further studies on the glycosylation of dysadherin will contribute to developing a strategy to inhibit cancer cell migration and invasion by dysadherin.

Overall, we first investigated the interrelationship of dysadherin and cell motility dynamics at single-cell resolution; the results showed that dysadherin is a molecular mechanics cue for cancer progression via actin dynamics and FAK signaling. These intriguing findings regarding dysadherin as a regulator of single-cell invasion highlight its significance in cell mechanics and progression, underscoring its potential as a therapeutic target in CC treatment.

## Supplementary Material

Supplementary figures and tables; video legends.

Supplementary videos 1-4.

Supplementary videos 5-8.

Supplementary videos 9-12.

Supplementary videos 13-16.

## Abbreviations

CC: colon cancer
FAK: focal adhesion kinase
FA: focal adhesion
KO: knockout
DEG: differentially expressed gene
GSEA: gene set enrichment analysis
UMAP: uniform manifold approximation and projection
EV: empty vector
OE: overexpression
IF: immunofluorescence
scRNA-seq: single-cell RNA sequencing
CRC: colorectal cancer
Dys: dysadherin
EMT: epithelial-mesenchymal transition
MLC2: myosin light chain 2
IC_50_: half-maximal inhibitory concentration
PTK: protein-tyrosine kinase
gRNA: guide ribonucleic acid
DNA: deoxyribonucleic acid
PCR: polymerase chain reaction
RIPA: radioimmunoprecipitation assay
BCA: bicinchoninic acid
SDS: sodium dodecyl sulfate
PAGE: polyacrylamide gel electrophoresis
PBS: phosphate-buffered saline
DAPI: 4',6'-diamidino-2-phenylindole
