# Supplementary material for: The dysadherin/FAK axis promotes individual cell migration in colon cancer
Source: Int J Biol Sci. 2024 Apr 8;20(7):2356–69. doi: 10.7150/ijbs.86699 (PMC11077383; doi:10.7150/ijbs.86699)
Supplement: Supplementary file 1 — Supplementary figures and tables; video legends. [file ijbsv20p2356s1.pdf]

Supplementary materials for

The dysadherin/FAK axis promotes individual cell migration in colon cancer

Choong-Jae Lee[1#], Tae-Young Jang[1#], Jee-Heun Kim[1#], Songwon Lim[1], Sunjae Lee[1], Jeong-Seok Nam[1]*

[1] School of Life Sciences, Gwangju Institute of Science and Technology, Gwangju 61005, Republic of Korea

* Corresponding author

Jeong-Seok Nam, School of Life Sciences, Gwangju Institute of Science and Technology, Gwangju, 61005, Republic of Korea; Email: namje@gist.ac.kr; Tel: +82 62 715 2893

# These authors contributed equally to this work

Supplementary Figures

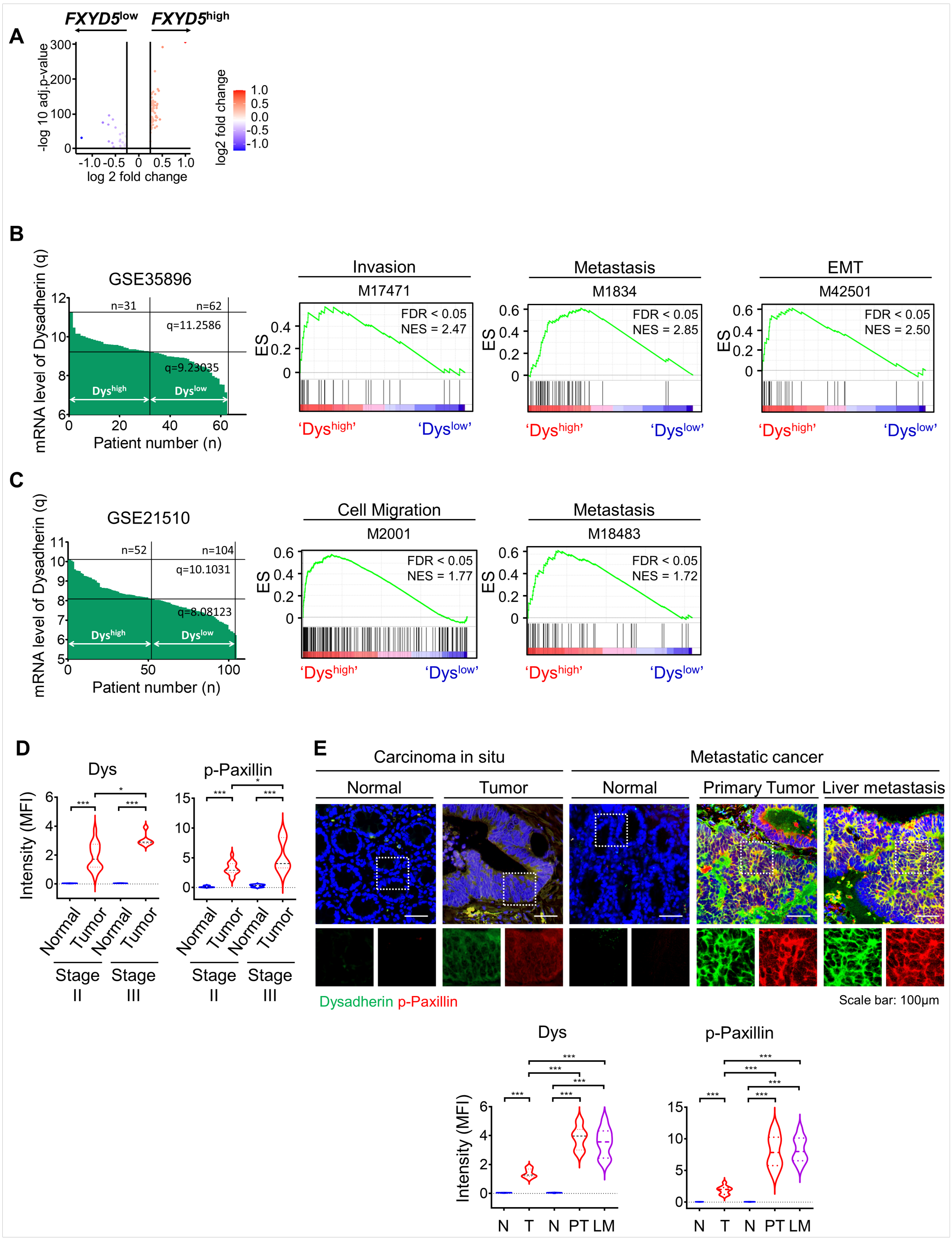

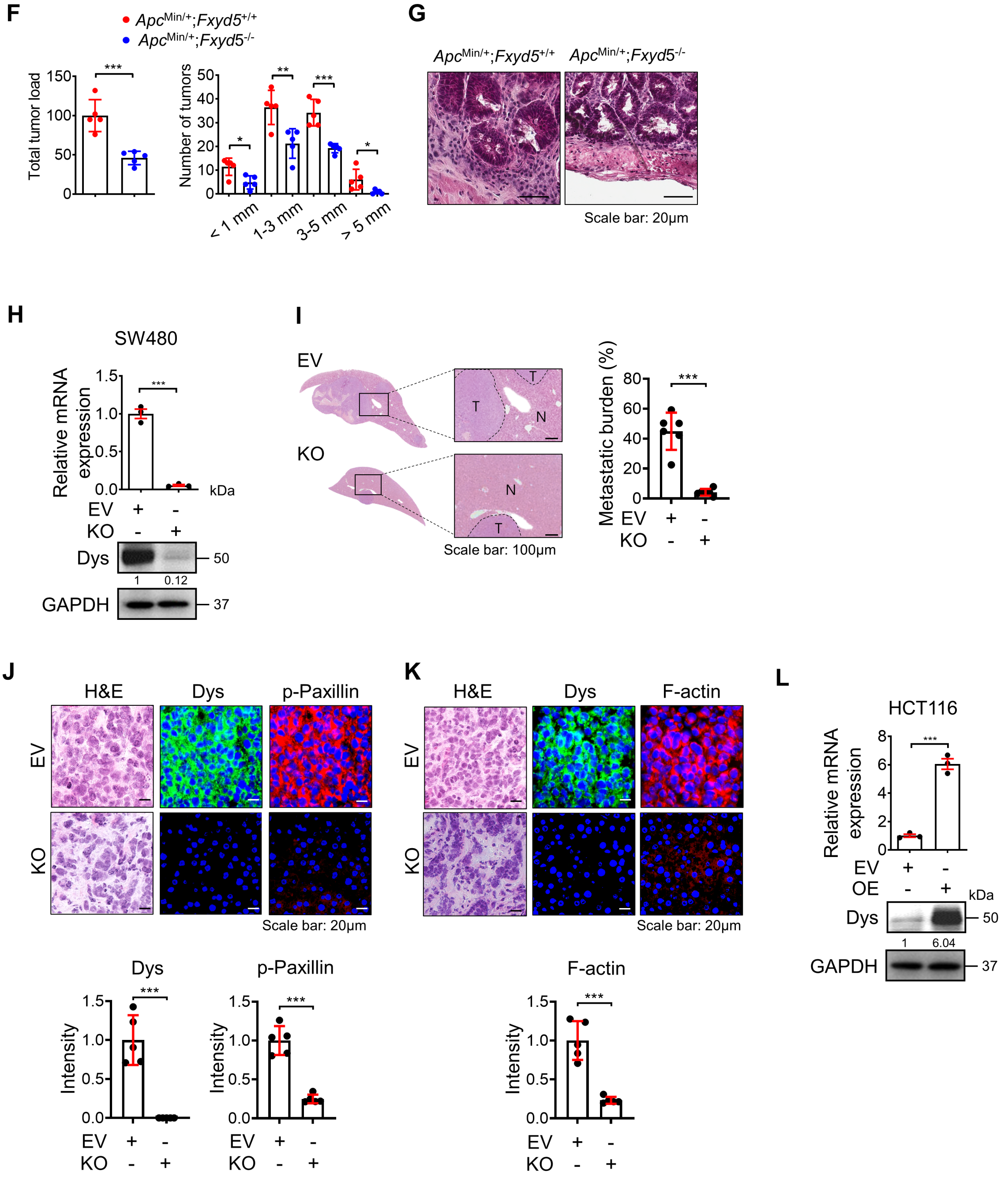

Figure S1. High dysadherin expression in cancer cells is correlated with cell migration and invasion. (A) Differentially expressed genes between the high and low dysadherin (Fxyd5)-expression cell groups. (B, C) GSEA of pathways enriched in dysadherin^{high} patients compared to dysadherin^{low} patients from open-source CRC patient data (GSE35896, GSE21510). (D) Quantitative analysis of immunofluorescence data for dysadherin and p-paxillin in CRC patient samples. (E) Immunofluorescence analysis of dysadherin and p-paxillin expression using CRC patient tissue. The patient number used in representative images is 17129828 for carcinoma in situ and 07115576 for metastatic CRC. Scale bars, 100 μm. N, normal; T, tumor; PT, primary tumor; LM, liver metastasis; MFI, mean fluorescence intensity. (F) Total tumor load and number of tumors distributed by size from *Apc*$^{Min/+}$;*Fxyd5*$^{+/+}$ and *Apc*$^{Min/+}$;*Fxyd5*$^{-/-}$ mice. (G) Representative hematoxylin and eosin-stained intestine with or

without invasive regions. (H) Altered expression of dysadherin analyzed by relative mRNA expression (top) and protein (bottom) levels upon dysadherin KO in SW480 cells. (I) Representative hematoxylin and eosin-stained livers with metastasis. N: normal, T: tumor. Quantitative analysis of metastatic burden at the microscopic level. Metastatic burden (%) = (Metastasized lesion area/Total tissue area) x 100. (J, K) Immunofluorescence analysis of dysadherin, F-actin and p-paxillin expression in metastatic nodules generated from invaded dysadherin EV or KO SW480 cells accompanied by H&E staining. (L) Altered expression of dysadherin analyzed by relative mRNA expression (top) and protein (bottom) levels upon dysadherin OE in HCT116 cells. The data are presented as the means ± SEMs. *** indicates $p < 0.001$. Statistical significance was determined by unpaired two-tailed Student's t tests for comparisons between two groups.

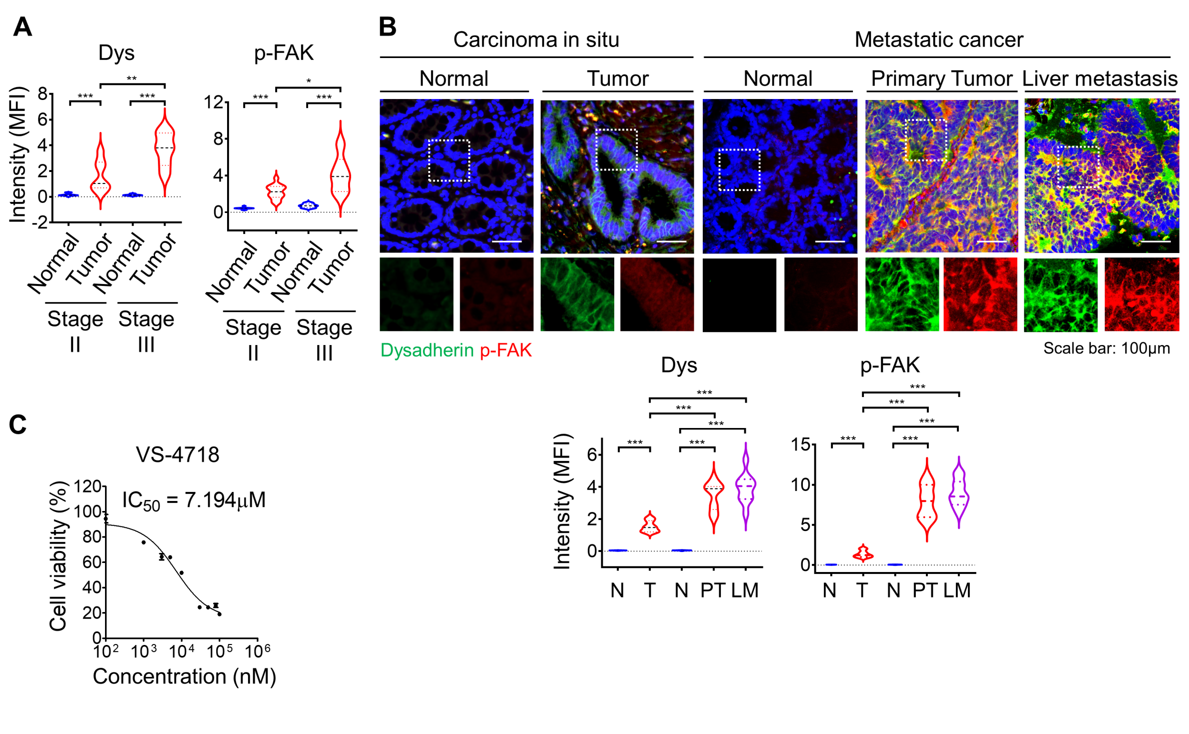

Figure S2. FAK expression in patient sample and validation of the cytotoxicity of VS-4718. (A) Quantitative analysis of dysadherin and p-FAK immunofluorescence data in patient samples. (B) Immunofluorescence analysis of dysadherin and p-FAK expression using CRC patient tissue. The patient number used in representative images is 09538529 for carcinoma in situ and 07115576 for metastatic CRC. Scale bars, 100 μm. N, normal; T, tumor; PT, primary tumor; LM, liver metastasis; MFI, mean fluorescence intensity. (C) The IC_{50} of VS-4718 was evaluated in HCT116 cells by the MTT assay.

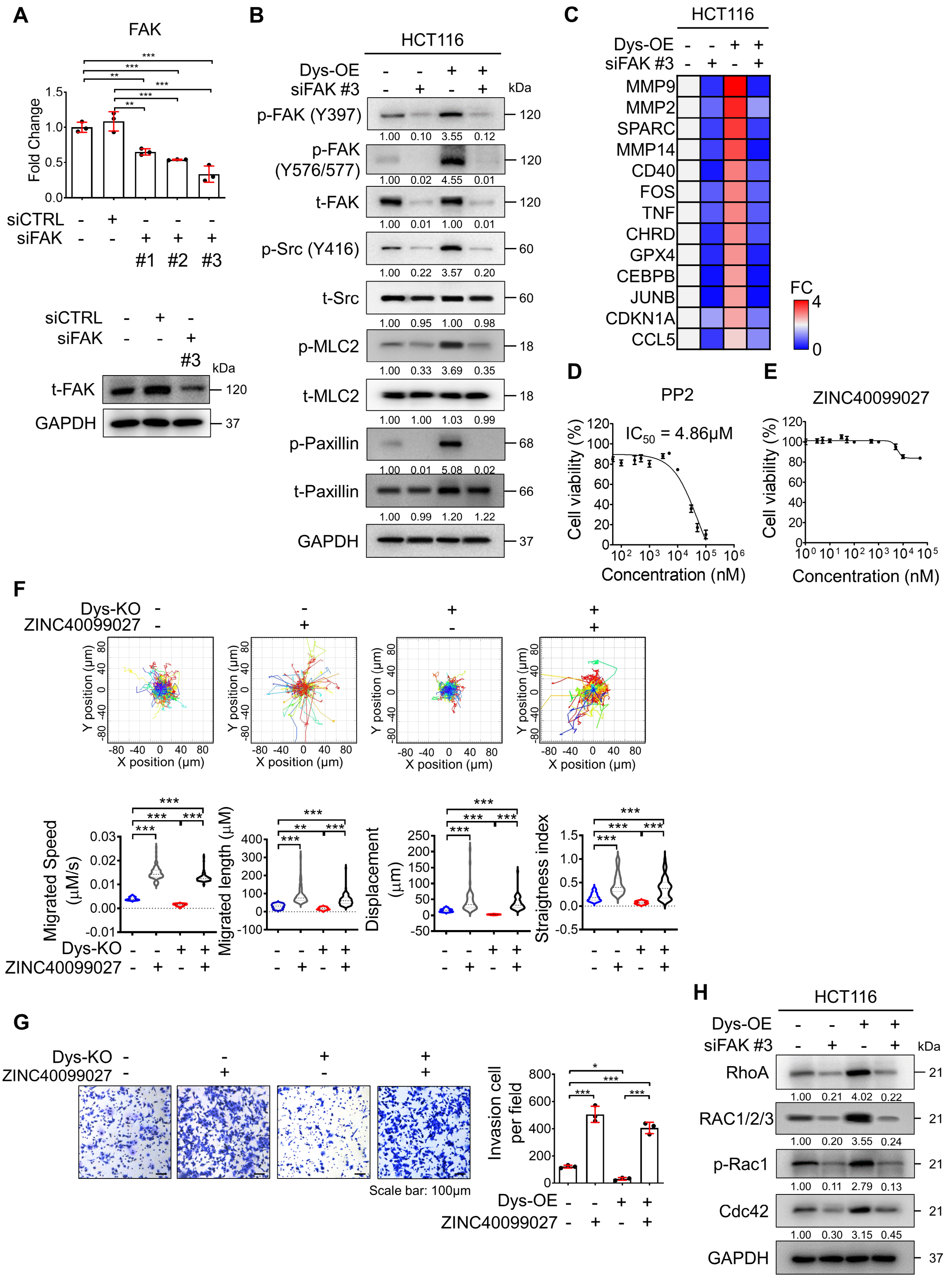
A
FAK
Fold Change
siCTRL
siFAK
#1 #2 #3
t-FAK
GAPDH
kDa
B
HCT116
Dys-OE
siFAK #3
p-FAK (Y397)
p-FAK (Y576/577)
t-FAK
p-Src (Y416)
t-Src
p-MLC2
t-MLC2
p-Paxillin
t-Paxillin
GAPDH
C
HCT116
MMP9
MMP2
SPARC
MMP14
CD40
FOS
TNF
CHRD
GPX4
CEBPB
JUNB
CDKN1A
CCL5
FC
D
PP2
IC50 = 4.86μM
Cell viability (%)
Concentration (nM)
E
ZINC40099027
F
Dys-KO
ZINC40099027
Y position (μm)
X position (μm)
Migrated Speed (μM/s)
Migrated length (μM)
Displacement (μm)
Straigtness index
G
Scale bar: 100μm
Invasion cell per field
H
HCT116
RhoA
RAC1/2/3
p-Rac1
Cdc42
GAPDH

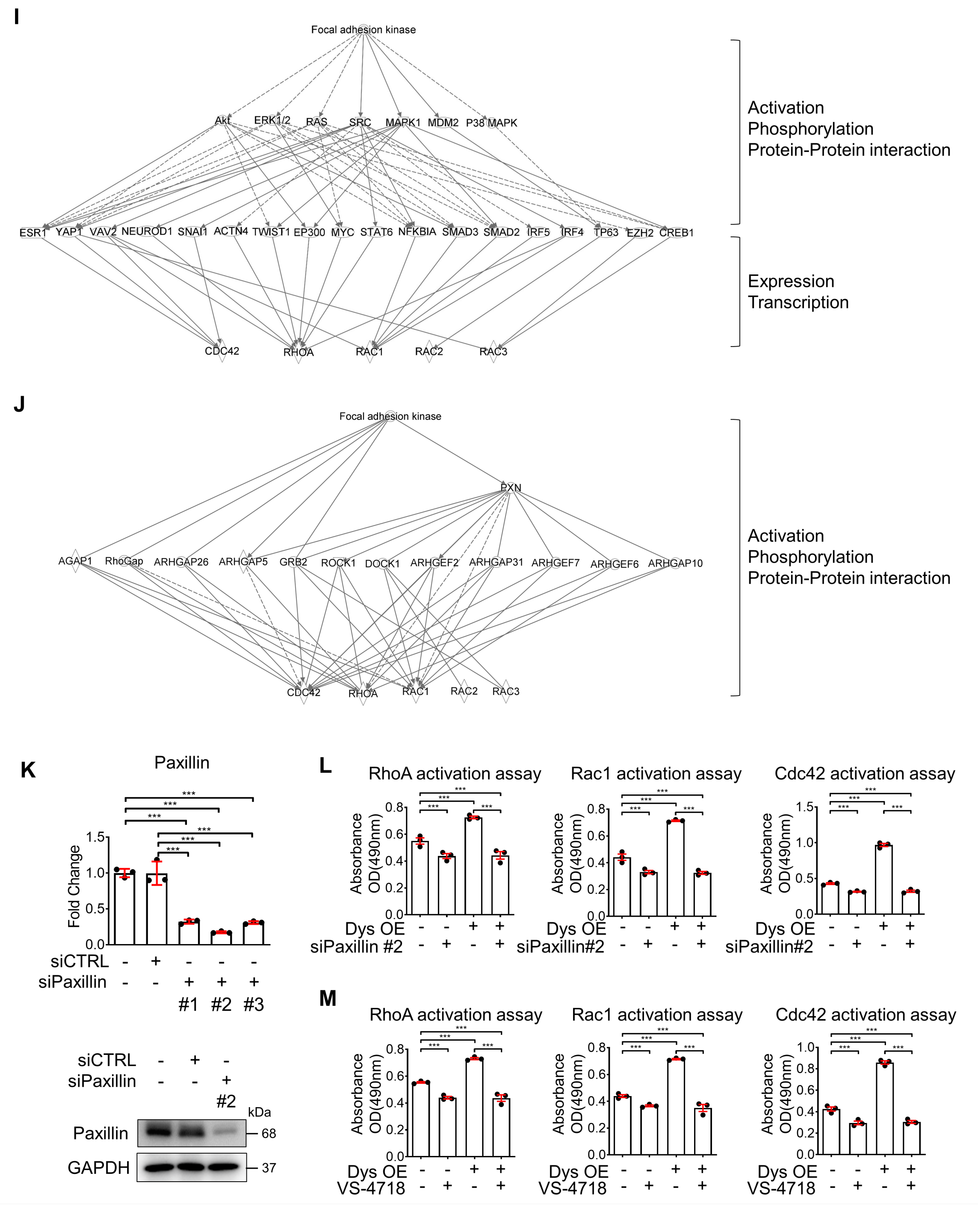

Figure S3. Validation of the cytotoxicity of VS-4718, ZINC40099027 and siRNA efficacy, and downstream signaling of dysadherin/FAK axis. (A) The efficiencies of three siFAK sequences were evaluated by real-time qPCR analysis (top) and immunoblot assay (bottom) in HCT116 cells. (B) Immunoblot of p-FAK, t-FAK, p-Src, t-Src, p-MLC2, t-MLC2, p-paxillin, and t-paxillin in dysadherin EV and OE HCT116 cells with or without siRNA targeting FAK. (C) Heatmap comparing the relative expression of FAK downstream genes in dysadherin EV and OE HCT116 cells with siFAK, as determined by RT–qPCR (n = 3 biological replicates). (D) The IC_{50} of PP2 was

evaluated in HCT116 cells by MTT assay. (E) The IC_{50} of ZINC40099027 was evaluated in SW480 cells by MTT assay. (F) Trajectories of individual cells (n=100) in a single field of view over an 8 h period in dysadherin EV and OE HCT116 cells with or without siRNA targeting FAK. Scatter plots showing the migrated length (μM), migrated speed (μM/s), straightness index and directionality. (G) Boyden chamber assays with Matrigel matrix-coated membranes were performed to compare the invasion potential of dysadherin EV and OE HCT116 cells with or without siRNA targeting FAK (n = 3/group). (H) Immunoblot of Rho-GTPase proteins in dysadherin EV and OE HCT116 cells with or without siRNA targeting FAK. (I) Pathway analysis by ingenuity pathway analysis of the expression of Rho-GTPase proteins induced by FAK signaling. (J) Pathway analysis by ingenuity pathway analysis of the activation of Rho-GTPase proteins induced by FAK signaling (K) The efficacy of three siPaxillin sequences was evaluated by real-time qPCR analysis (top) and immunoblot assay (bottom) in HCT116 cells. (L, M) The activation of Rho-GTPase proteins (RhoA, Rac1, and Cdc42) was measured by G-LISA assay in dysadherin EV and OE HCT116 cells with or without 1 μM VS-4718 treatment or siRNA targeting paxillin.

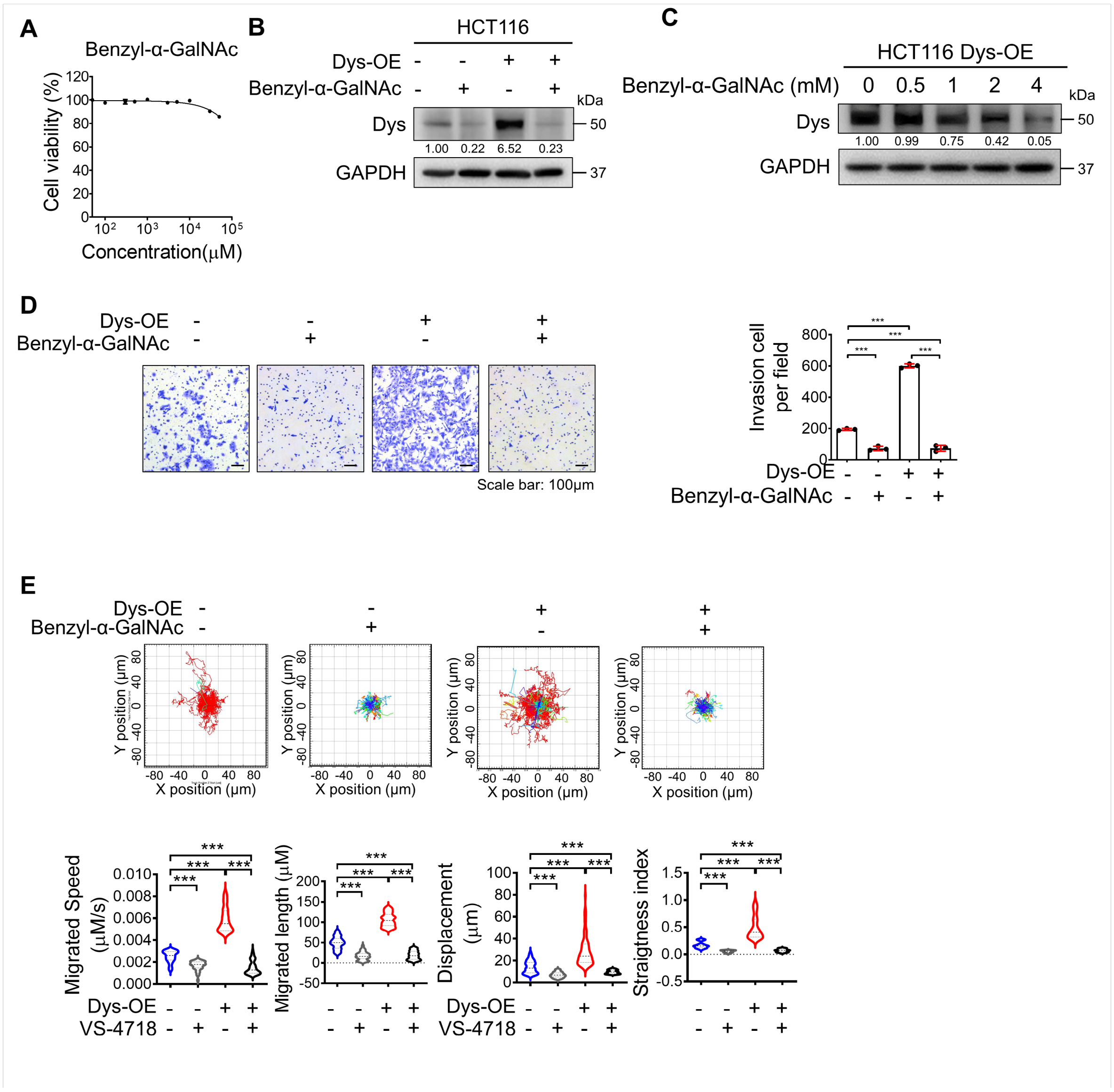

Figure S4. The effects of glycosylation inhibition on the expression and function of dysadherin. (A) The IC_{50} of benzyl-α-GalNAc was evaluated in HCT116 cells by MTT assay. (B) Immunoblot of dysadherin and GAPDH in dysadherin EV and OE HCT116 cells with or without 4 mM benzyl-α-GalNAc treatment. (C) Immunoblot of dysadherin and GAPDH in dysadherin OE HCT116 cells with 0, 0.5, 1, 2, 4 mM benzyl-α-GalNAc treatment. (D) Transwell invasion assays with Matrigel matrix-coated membranes were performed to compare the invasion potential of WT HCT116 and dysadherin OE HCT116 cells with or without 4 mM benzyl-α-GalNAc (n = 3/group). (E) Trajectories of individual cells (n=100) in a single field of view over an 8-h period upon 4 mM benzyl-α-GalNAc treatment. Scatter plots show the migrated length (μM), migration speed (μM/s), straightness index and directionality.

Supplementary Videos

Video S1. Representative single-cell tracking video in control SW480 cells.

Video S2. Representative single-cell tracking video in SW480 cells with dysadherin knockout.

Video S3. Representative single-cell tracking video in control HCT116 cells.

Video S4. Representative single-cell tracking video of HCT116 cells overexpressing dysadherin.

Video S5. Representative single-cell tracking video in control HCT116 cells.

Video S6. Representative single cell-tracking video in HCT116 cells treated with VS-4718

Video S7. Representative single-cell tracking video of HCT116 cells overexpressing dysadherin.

Video S8. Representative single-cell tracking video of HCT116 cells overexpressing dysadherin treated with VS-4718.

Video S9. Representative single-cell tracking video in control HCT116 cells.

Video S10. Representative single cell-tracking video in HCT116 cells treated with benzyl-α-GalNAc

Video S11. Representative single-cell tracking video of HCT116 cells overexpressing dysadherin.

Video S12. Representative single-cell tracking video of HCT116 cells overexpressing dysadherin treated with benzyl-α-GalNAc.

Video S13. Representative single-cell tracking video in control SW480 cells.

Video S14. Representative single cell-tracking video in SW480 cells treated with ZINC40099027

Video S15. Representative single-cell tracking video of SW480 cells with dysadherin knockout.

Video S16. Representative single-cell tracking video of SW480 cells with dysadherin knockout treated with ZINC40099027.

Supplementary Tables

Table S1. List of primers used for real-time RT–qPCR

Target	Sequence	
	Forward	Reverse
Human Dysadherin	TCCCACTGATGACACCACGA	AAACCAGATGGCTTGAGGGT
Human NOS3	TGACATTGAGAGCAAAGGGCT	CGTAGGTCTTGGGGTTGTCA
Human BEX4	AGAATCCCGCCATTTGGGA	TTCCATCATCTGCCCTACAAACC
Human TPGS1	GCCTTCCTGGCTCACTACTT	CACGCTCACGTTGTTGTTGA
Human FGD5	TGCCTGTCACACACGTCA	TGTCCTTCAGGTACTTCAGCG
Human FLNB	GGGGCACCACAGGTATCCA	CCATGGGGGTGTACATGACTTT
Human ITGA2B	ATCAGTTTGTGCTGCAGTCG	ACCAGATTGGAATGGCCCTC
Human ITGB2	GGATGGACCGCTACCTCATC	TCTCAAAGCGCCTGTACTCC
Human EFNB1	TCCTGACGGTCCTACTACTGA	AGGGAATGATGATGTCGCTGG
Human CCR7	ACGGTGGCCAACTTCAACAT	GATCGTTGCGGAACTTGACG
Human CLDN11	GAGCCCGGTGTGGCTAAGTA	GCCTGCATACAGGGAGTAGC
Human CXCL6	TGCGTTGCACTTGTTTACGC	TTTAGAAAAGGGGCTTCCGGG
Human ACVRL1	ACCATCGTGAATGGCATCG	CTGCAGCCAGCCGGTTA
Human CXCL8	GAGAGTGATTGAGAGTGGACCAC	CACAACCCTCTGCACCCAGTTT
Human CCL11	GGGTGCAGGATTCCATGAAGTA	AAACCCATGCCCTTTGGACTG
Human CXCR1	TGAGCGCCGCAACAACAT	CGTGCCAAGAACTCCTTGCT
Human CAMK1	TGAACAGATTTTGAAGGCCGAG	CAATCCATGGGTGCTGCAAG
Human Paxillin	CTTTCTGAACTCGACCGCCT	TCCCAAGGGGCTGTTAGTCT
Human PPIA	TGCCATCGCCAAGGAGTAG	TGCACAGACGGTCACTCAAA
Human SPARC	AGACAGGGGTACCTGTGGG	CACATGGGGGTGTTGCTCTC
Human MMP2	AAGGATGGCAAGTACGGCTT	AAACTTGCAGGGCTGTCCTT
Human CD40	AATGCCTTCCTTGCGGTGAA	TCTCACAGGCCTCACTCGTA
Human CCL5	TCAAGACAGCACGTGGACCT	CGGGCAATGTAGGCAAAGCA
Human MMP9	GTCGAAATCTCTGGGGCCTG	ATGTTGTGGTGGTGCCACTT
Human TNF	CAGGCAGGTTCTCTTCCTCTCA	AGGAGAAGAGGCTGAGGAACAA
Human	CAGACTACGAGGCGTCATCC	CGTGGGAATGAAGTTGGCAC

FOS		
Human GPX4	ACGCCCGATACGCTGAGT	TCACGCAGATCTTGCTGAACATA
Human MMP14	CCTTGGACTGTCAGGAATGAGG	TTCTCCGTGTCCATCCACTGGT
Human CHRD	CGCATCAGTGGACACATTGC	CCTCACTGCTTGTCCCTACC
Human CDKN1A	ACTTTGTCACCGAGACACCA	CAGCAGAGCAGGTGAGGTG
Human CEBPB	TGATAAACTCTCTGCTTCTCCCT	GTTGCGTCAGTCCCGTGT
Human JUNB	ACCACGACGACTCATACACAG	CGAGCCCTGACCAGAAAAGT

Table S2. List of antibodies used for immunoblotting analysis and IF staining

Immunoblot analysis

Target	Conjugate	Catalog# (company or provider)
Human p-MLC (Ser19)	-	3675/Cell Signaling Technology
Human t-MLC	-	8505/Cell Signaling Technology
Human p-Paxillin (Tyr118)	-	69363/Cell Signaling Technology
Human t-Paxillin	-	2542/Cell Signaling Technology
Human Vinculin	-	13901/Cell Signaling Technology
Human p-FAK	-	3283/Cell Signaling Technology
Human T-FAK	-	3285/Cell Signaling Technology
Human p-Src	-	59548/Cell Signaling Technology
Human T-Src	-	2109/Cell Signaling Technology
Human RhoA	-	2117/Cell Signaling Technology
Human Rac1/2/3	-	2465/Cell Signaling Technology
Human p-Rac1/cdc42 (Ser71)	-	2461/Cell Signaling Technology
Human Cdc24	-	2460/Cell Signaling Technology
Human Dysadherin	-	M53/provided by Dr. Ino
Human GAPDH	-	5174/Cell Signaling Technology
Goat anti-mouse	HRP	554002/BD Pharmigen™
Goat anti-rabbit	HRP	554021/BD Pharmigen™

IF staining

Target	Conjugate	Catalog# (company or provider)
Human Dysadherin	-	M53/provided by Dr. Ino
Human F-actin	Alexa 555	A34055/Thermo Fisher
Human p-MLC2	-	3675/Cell Signaling Technology
Human p-Paxillin	-	69363/Cell Signaling Technology
Human p-FAK (Y397)	-	700255/Thermo Fisher
Donkey anti-mouse	Alexa 488	A21202/Thermo Fisher
Goat anti-mouse	Alexa 555	A21422/Thermo Fisher
Donkey anti-rabbit	Alexa 488	A21206/Thermo Fisher
Donkey anti-rabbit	Alexa 555	A31572/Thermo Fisher

Table S3. List of siRNA targeting FAK

Target		Sequence	
		Sense	Anti-sense
FAK	#1	AACCACCUGGGCCAGUAUUAU	AUAAUACUGGCCCAGGUGGUU
	#2	GCGAUUAUAUGUUAGAGAUAGUU	AACUAUCUCUAACAUAUAAUCGC
	#3	CGAAUGAUAAGGUGUACGA	UCGUACACCUUAUCAUUCG
Paxillin	#1	GAUUUCUACACUGAGGUUU	AAACCUCAGUGUAGAAAUC
	#2	GCUCACCAGGACUGUUUUA	UAAAACAGUCCUGGUGAGC
	#3	CAGAACGACAAGCCUUACU	AGUAAGGCUUGUCGUUCUG

Table S4. Association of clinicopathologic characteristics in patients with resected CRC (n = 20)

	Stage		
Variable	Stage II (n=14)	Stage III (n=6)	*p* value
Age (mean±SD)	65.14±10.38	59.17±9.559	0.244
Sex			
Male	8 (57.1%)	3 (50.0%)	0.484
Female	6 (42.9%)	3 (50.0%)	
T stage			
T2-3	13 (89.6%)	5(70.4%)	0.515
T4	1 (10.4%)	1 (29.6%)	
N stage			
N0	14 (100.0%)	0 (0.0%)	**< 0.001**
N1	0 (0.0%)	2 (33.3%)	
N2	0 (0.0%)	4 (66.7%)	
Tumor differentiation			
Well	5 (35.7%)	0 (0%)	**0.049**
Moderately	8 (57.1%)	3 (50.0%)	
Poorly	1 (7.1%)	3 (50.0%)	
Recurrence			
Yes	1 (7.1%)	2 (25.0%)	0.240
No	13 (92.9%)	6 (75.0%)	

Table S5. Clinical information regarding the CRC patient samples used for IF staining

# of Patients	Site	Sex	Age	Cell metaplasia	AJCC Stage	TNM Stage	
						Tumor depth	Lymph node metastasis
71047123	colon	F	56	yes	Stage III	T3	N2a
71045602	colon	F	60	yes	Stage II	T3	N0
71040975	rectum	M	61	yes	Stage II	T3	N0
71038481	rectum	F	80	yes	Stage II	T3	N0
71037976	colon	M	64	yes	Stage II	T3	N0
71036347	rectum	M	68	yes	Stage II	T3	N0
71034747	rectum	M	62	yes	Stage II	T3	N0
71033802	rectum	F	56	yes	Stage III	T3	N2a
71030718	colon	M	50	yes	Stage III	T3	N1b
71029709	colon	M	49	yes	Stage II	T3	N0
71023619	colon	M	52	yes	Stage II	T3	N0
71013348	colon	M	62	yes	Stage III	T3	N1b
71003674	colon	F	73	yes	Stage II	T3	N0
70471102	colon	M	72	yes	Stage II	T3	N0
70327925	colon	M	61	yes	Stage II	T4a	N0
18301077	colon	F	52	yes	Stage II	T3	N0
15709306	colon	F	78	yes	Stage II	T3	N0
15120242	colon	F	80	yes	Stage II	T3	N0
14847893	colon	F	77	yes	Stage III	T4a	N2a
11841258	rectum	M	54	yes	Stage III	T3	N2b

Table S6. Association of clinicopathologic characteristics in patients with resected CRC (n = 20)

Variable	Carcinoma in situ (n=10)	Metastatic CRC (n=10)	*p* value
Age (mean±SD)	67±5.08	59.17±9.559	0.624
Sex			
Male	6 (60.0%)	7 (70.0%)	0.639
Female	4 (40.0%)	3 (30.0%)	
T stage			
Tis	1 (10.0%)	0 (0.0%)	0.098
T1	3 (30.0%)	0 (0.0%)	
T2-3	6 (60.0%)	8 (80.0%)	
T4	0 (0.0%)	2 (20.0%)	
N stage			
N0	10 (100.0%)	4 (40.0%)	**0.014**
N1	0 (0.0%)	3 (30.0%)	
N2	0 (0.0%)	3 (30.0%)	
Tumor differentiation			
Well	4 (40.0%)	3 (30%)	0.639
Moderately	6 (6001%)	7 (70.0%)	
Poorly	0 (0.0%)	0 (0.0%)	

Table S7. Clinical information regarding the CRC patient samples used for IF staining

# of Patients	Site	Sex	Age	Cell metaplasia		TNM Stage	
						Tumor depth	Lymph node metastasis
17129828	colon	M	67	yes	Carcinoma in situ	T1	N0
71010056	colon	M	69	yes	Carcinoma in situ	T1	N0
71030393	rectum	F	70	yes	Carcinoma in situ	T2	N0
09538529	rectum	M	60	yes	Carcinoma in situ	Tis	N0
11647306	rectum	F	77	yes	Carcinoma in situ	T2	N0
71024165	colon	M	63	yes	Carcinoma in situ	T3	N0
71040258	colon	F	68	yes	Carcinoma in situ	T2	N0
71040067	rectum	F	69	yes	Carcinoma in situ	T1	N0
13524348	colon	M	60	yes	Carcinoma in situ	T2	N0
71048234	colon	M	67	yes	Carcinoma in situ	T2	N0
07506122	colon	M	64	yes	Metastatic CRC	T3	N0
07427312	rectum	M	68	yes	Metastatic CRC	T4a	N1a
07115576	colon	F	55	yes	Metastatic CRC	T4a	N2b
07432965	rectum	M	73	yes	Metastatic CRC	T3	N0
07076544	colon	M	59	yes	Metastatic CRC	T3	N0
07246138	colon	F	62	yes	Metastatic CRC	T3	N2b
07593199	colon	M	64	yes	Metastatic CRC	T3	N1b
07126837	colon	M	84	yes	Metastatic CRC	T3	N0
07620184	colon	M	86	yes	Metastatic CRC	T3	N1a
07617821	colon	F	73	yes	Metastatic CRC	T3	N2a
